# Acute effects of three warm-up protocols on drop jump biomechanics in elite Taekwondo athletes: An IMU-based analysis

**DOI:** 10.1371/journal.pone.0351884

**Published:** 2026-06-22

**Authors:** Somayeh Taghidoust Chahardeh, Seyyed Hossein Hosseini, Ali Shamsi Majelan

**Affiliations:** 1 MSc in Sports Biomechanics, Faculty of Physical Education and Sport Sciences, University of Guilan, Rasht, Guilan, Iran; 2 Assistant Professor in Sports Biomechanics, Department of sports sciences, Faculty of Physical Education and Sport Sciences, University of Guilan, Rasht, Guilan, Iran; 3 Associate Professor in Corrective Exercise, Department of sports injuries & corrective exercise, Faculty of Physical Education and Sport Sciences, University of Guilan, Rasht, Guilan, Iran; Federation University Australia, AUSTRALIA

## Abstract

This randomized, parallel-group pre–post study examined the acute effects of three warm-up modalities on stretch–shortening cycle (SSC) biomechanics in elite female Taekwondo athletes. Thirty-six participants were randomly assigned to dynamic stretching, foam rolling, or resistance-band (TheraBand) protocols following a standardized submaximal treadmill warm-up. Three-dimensional kinetic and temporal variables were collected during drop jump tasks before and after each intervention using inertial measurement units.Between-group post-intervention differences were analyzed using analysis of covariance (ANCOVA) or rank-based Quade’s ANCOVA, controlling for baseline disparities. Within-group changes were assessed using paired parametric or non-parametric tests as appropriate. Effect sizes (η²) are reported alongside p-values to indicate the magnitude of observed effects.Significant group effects were observed for concentric phase duration (η² = 0.177), peak concentric force (η² = 0.186), and mean concentric power (η² = 0.211). Post-hoc analyses revealed that dynamic stretching enhanced concentric force and power, while foam rolling primarily altered temporal characteristics of force development. TheraBand preserved performance close to baseline. In contrast, gross performance outcomes such as jump height, flight time, and contact time showed trivial effect sizes (η² ≤ 0.104) and no significant between-group differences. These findings indicate that warm-up modalities selectively modulate force- and power-related components of SSC function rather than uniformly enhancing overall jump performance. By ‘selective modulation,’ we mean that different warm-up protocols influence distinct components of stretch-shortening cycle function rather than producing a generalized enhancement. Specifically, dynamic stretching primarily improved concentric force and power output; foam rolling altered temporal characteristics (e.g., concentric duration and time to peak power) without increasing peak force; and TheraBand preserved baseline performance across most variables. This selective response indicates that warm-up modality should be matched to the specific neuromuscular demand of the subsequent task. Post-hoc comparisons clarify which groups differ, highlighting the practical utility of dynamic stretching for enhancing propulsive-phase mechanics in elite athletes.

## Introduction

Warming up is a fundamental component of athletic preparation and is widely implemented to enhance performance and reduce injury risk. Previous studies have shown that different warm-up protocols can exert distinct effects on neuromuscular and biomechanical performance [1–3]. Among these methods, dynamic warm-up —defined as active movements that take joints and muscles through a full range of motion in a controlled, repetitive manner (e.g., leg swings, lunges, high knees, and skipping)— is widely recognized for its efficacy in boosting muscular performance and increasing joint range of motion. By elevating muscle temperature and improving blood flow, dynamic warm-up effectively prepares the body for intense physical exertion and is considered one of the most effective preparatory strategies [4,5].

An additional complementary element in warm-up protocols is external resistance training, such as resistance bands (Theraband), which serve as targeted tools for muscle activation and movement pattern refinement. Training with resistance bands has been shown to enhance muscular strength and improve kinematic control, factors particularly significant for taekwondo athletes who rely on rapid and explosive movements [6,7]. Furthermore, foam rolling, applied as a self-myofascial release (SMR) technique, has gained popularity in recent years. Research indicates that foam rolling can increase range of motion and reduce muscle stiffness [8,9]. However, findings regarding its impact on biomechanical performance remain inconclusive and appear contingent on implementation specifics and duration.

Taekwondo is a discipline characterized by rapid strikes, explosive jumps, and swift directional changes, where competitive success heavily depends on the ability to generate and absorb high forces in short periods [10,11].A key biomechanical feature underlying these actions is the stretch-shortening cycle (SSC), which enables muscles to store and release elastic energy during rapid eccentric-concentric transitions. The drop jump (DJ) is a widely used plyometric task to assess stretch-shortening cycle (SSC) function, reflecting an athlete’s ability to rapidly absorb and reutilize elastic energy during the landing-to-takeoff phase [12]. During the DJ, biomechanical parameters such as joint torques, power output, ground reaction forces, and ground contact time provide insight into an athlete’s capacity for rapid force generation and neuromuscular control [13–15]. Therefore, analyzing DJ performance offers a valid and controlled model to evaluate explosive lower-limb function relevant to Taekwondo-specific actions. The drop jump’s capacity to exploit elastic energy and reactive strength contributes to increased power and speed of strikes, fundamental factors in scoring during competitions [16].

Moreover, studies utilizing inertial measurement units (IMU) in taekwondo athletes have demonstrated that more precise kinetic and kinematic patterns during drop jumps associate with superior technical execution and reduced injury risk. These findings highlight the paramount importance of biomechanical assessment in training monitoring [17].

Finally, warm-up protocols that influence drop jump performance induce significant alterations in joint torques and neuromuscular readiness, underscoring the need for specifically designed warm-up strategies in elite taekwondo athletes to enhance performance and mitigate injury risk [2,17].

While the individual effects of dynamic stretching, foam rolling, and resistance band exercises have been explored separately in various athletic populations, a direct comparison of their acute biomechanical impacts is lacking. This gap is especially pertinent for elite Taekwondo athletes, whose performance relies on explosive, stretch-shortening cycle-dependent movements. Therefore, this study uniquely juxtaposes these three common warm-up modalities to determine their immediate efficacy in optimizing kinetic and kinematic performance during a drop jump task in this specific cohort.

Taekwondo performance depends heavily on rapid hip flexion, knee extension, and controlled landing following explosive movements such as jumping kicks. Therefore, optimizing pre-performance readiness through warm-up routines that closely mimic these technical demands is essential. The present study aims to compare the acute effects of three warm-up protocols—dynamic stretching, foam rolling, and resistance band exercises—on kinetic and kinematic variables during drop jumps in elite Taekwondo practitioners.This approach helps evaluate how each warm-up strategy influences sport-relevant neuromechanical performance. We hypothesized that: (1) dynamic stretching would yield the greatest improvement in power-related variables due to its neuromuscular activation properties; (2) resistance band warm-ups would enhance force production and impulse; and (3) foam rolling would potentially dampen explosive performance metrics due to its potential to reduce muscle-tendon stiffness acutely.

Although the drop jump is not a Taekwondo-specific technique, it was selected as a valid and controlled biomechanical model that isolates the explosive stretch-shortening cycle action fundamental to Taekwondo kicking, allowing for precise measurement of warm-up effects without the technical variability of a full kick.

## Materials and Methods

This study was approved by the Ethics Committee of the University of Guilan (IR.GUILAN.REC.1403.127). All procedures performed were in accordance with the ethical standards of the institutional research committee and with the 1964 Helsinki declaration and its later amendments. Written informed consent was obtained from all individual participants included in the study.

The following sections describe the participants, study design, measurement procedures (kinetic and Kinematic Data Acquisition, and signal processing), warm-up protocols, and statistical analyses in sequential order.

### Participants

Based on an a priori power analysis using G*Power software (version 3.1.9.4) for an ANCOVA design accounting for within- and between-group interactions, a minimum sample size of 12 participants per group was required to achieve 80% statistical power at an alpha level of 0.05, assuming a medium effect size (f = 0.25) and an intraclass correlation of 0.5 [18]. The calculation was informed by prior studies investigating the acute effects of warm-up interventions on plyometric and stretch–shortening cycle performance [4,8].Consequently, 36 elite female Taekwondo athletes (age 18–25 years, black belt or higher, average training experience >10 years, no musculoskeletal injuries in the past five years) were randomly assigned to one of three warm-up groups (Foam roll, Dynamic stretching, Theraband) using a computer-generated random sequence. Randomization was performed by an independent researcher who was blinded to data collection to minimize allocation bias.Inclusion criteria were: a minimum of five years of competitive Taekwondo experience, maintenance of high-level athletic performance, female gender, absence of injury or musculoskeletal disorders in the past five years, no drug dependency or health problems, and abstention from strenuous exercise for 48 hours prior to testing while maintaining optimal physical readiness. Participants were also tested outside of their menstrual period to avoid hormonal variability. Exclusion criteria included prior lumbar or lower-limb surgeries, skeletal-muscular abnormalities, or orthopedic disorders [19]. Participants who missed any pre- or post-test session, withdrew, or experienced pain or injury during testing were excluded.All participants received detailed information about the study objectives, procedures, and potential risks. Written informed consent was obtained prior to participation. Anthropometric characteristics and training background of the participants are presented in Table 1.

**Table 1:** Baseline anthropometric and training characteristics of the participants by experimental group.

Parameter	Group	Mean	SD	F-stat	p-value
Age (years)	Foam Roll	21.55	2.18	0.01	0.982
	Dynamic	21.50	1.85		
	Theraband	21.37	1.84		
Height (cm)	Foam Roll	164.50	13.11	0.28	0.756
	Dynamic	165.43	15.80		
	Theraband	166.25	14.25		
Weight (kg)	Foam Roll	58.36	5.76	0.45	0.639
	Dynamic	57.56	6.16		
	Theraband	60.43	6.75		
BMI (kg/m²)	Foam Roll	21.58	2.29	0.38	0.687
	Dynamic	20.99	1.95		
	Theraband	21.86	2.20		
Training (hours/week)	Foam Roll	12.5	1.45	0.33	0.961
	Dynamic	13.1	1.69		
	Theraband	12.8	1.75		

### Study design

A randomized, parallel-group, repeated-measures (pre–post) experimental design was employed. The study was assessor-blinded: the researcher responsible for processing and analyzing the IMU data was blinded to group allocation throughout the study. The primary objective of the study was to compare the effects of three warm-up protocols—foam rolling, dynamic stretching, and resistance band exercises—on kinetic and kinematic variables during the execution of a drop jump task in elite Taekwondo athletes.

All assessments were conducted within a single 80-minute testing session under standardized laboratory conditions (see Table 2 for the complete experimental timeline). Testing was uniformly scheduled between 10:00 AM and 2:00 PM throughout the autumn season to control for potential diurnal variations. The laboratory temperature was maintained at 22 ± 2°C. Additionally, to control for biological variability, all female participants were confirmed not to be in their menstrual phase during testing. The methodological rigor applied in the present study provided a valid framework for comparing the three warm-up protocols under controlled conditions.

**Table 2:** Experimental timeline of the single 80-minute testing session.

Phase	Duration	Activity
1	20 min	Arrival, informed consent, anthropometric measurements, IMU sensor attachment and calibration, task familiarization
2	10 min	Standardized submaximal warm-up: treadmill walking (5 min at 4 km/h) + running (5 min at 7 km/h)
3	3 min	IMU sensor attachment
4	6-8 min	Pre-test assessment: 3 drop jumps (60 sec rest between jumps)
5	10-12 min	Experimental warm-up intervention (randomized: Dynamic Stretching / TheraBand / Foam Rolling), including explanation, rest intervals, and individual execution differences
6	6-8 min	Post-test assessment: 3 drop jumps performed immediately after intervention (60 sec rest between jumps)
7	20 min	Cool-down, light stretching, sensor removal

### Kinetic and kinematic data acquisition

Three-dimensional motion data were collected using an inertial measurement unit (IMU) system (model: Mocap-Apex). The system’s accuracy was validated against a force plate during drop jumps, showing high intraclass correlation (ICC > 0.90) for key parameters. Raw data were sampled at 1000 Hz. Full technical specifications are provided in S1 Table in S1 Dataset. The validity of IMU-based jump assessment has been further supported by recent studies comparing inertial sensors with gold-standard force plate measurements [20–22].

Real-time monitoring of data quality and integrity was conducted using the proprietary Apex software during both recording and analysis stages; any detected artifacts were reviewed and corrected as necessary. Four IMU modules were strategically attached to the dominant lower limb of each participant to collect kinetic and kinematic data. The placement sites were carefully chosen to minimize muscle mass interference while maximizing signal stability.

Specifically, the sensors were positioned as follows:

1. On the dorsal surface of the foot,
2. On the distal segment of the shank, just above the ankle joint,
3. On the distal region of the thigh, proximal to the knee joint,
4. On the posterior aspect of the pelvis (pelvic region).

Specialized adhesive straps were employed to secure the sensors firmly and prevent unwanted displacement during movement tasks.

The wearable IMU system used in this study has been previously validated for assessing vertical jump kinetics and kinematics, demonstrating high reliability and agreement with force plate measurements [20]. This supports the use of torso- and waist-mounted sensors to quantify kinetic and temporal parameters during explosive lower-limb tasks in athletes.

The biomechanical task assessed was a drop jump from a standardized 40 cm platform. Participants were instructed to jump vertically with maximal effort immediately upon ground contact. Data captured included contact time, duration of eccentric and concentric phases, jump height, flight time, and other relevant parameters, all recorded and processed using the Apex software with a standard sampling frequency.

### Processing and analysis of IMU data

All signal processing steps were performed within the APEX software environment. A fifth-order low-pass Butterworth filter with a cutoff frequency of 40 Hz was applied to the data to minimize external noise and artifacts. This filtering approach conforms to established standards for analyzing biomechanical signals of dynamic movements. The filtered data were segmented into distinct movement phases—eccentric and concentric—and key biomechanical parameters were extracted following international guidelines.

### Extraction of key parameters

Primary kinetic and temporal variables were extracted from IMU data using validated biomechanical methods [21,23,24] (Table 3).

**Table 3:** Definition and calculation of key biomechanical parameters extracted from IMU data.

Parameter	Definition / Calculation
**Flight time**	Interval from take-off to ground contact (threshold-based event detection)
**Jump height (h)**	h = (tF² × g) / 8, where tF = flight time, g = 9.81 m/s²
**Contact time**	Duration of ground contact (identified via vertical acceleration changes)
**RSImod**	Jump height (m) ÷ contact time (s)
**ECC / CON duration**	Eccentric phase: ground contact to concentric onset; Concentric phase: to take-off
**Peak / Mean Force**	Derived from acceleration × body mass; normalized to Nm/kg
**Peak / Mean Power**	Power = Force × Velocity; normalized to W/kg
**Impulse**	∫ F(t) dt (Newton-seconds), reported separately for ECC and CON phases
**Time to peak**	Time from phase onset to peak force/power (indicator of neuromuscular response speed)

All IMU modules were wirelessly synchronized. Automated quality control excluded segments with joint angle errors >5° or event delays >5 ms. IMU-derived parameters are indirect estimations and should be interpreted as approximations of muscular output rather than absolute mechanical quantities [20].

### Warm-up protocols

At the beginning of the session, participants performed a general submaximal warm-up lasting 10 minutes on a motorized treadmill (serial number 1005735). This protocol consisted of 5 minutes of walking at a constant speed of 4 km/h, followed by 5 minutes of running at 7 km/h. The sole purpose of this phase was to establish a common baseline of core and muscle temperature across all groups before implementing the experimental warm-up interventions. This standardization ensures that any subsequent differences between groups can be attributed solely to the experimental protocols rather than to pre-existing temperature disparities [3]. It is important to distinguish between core temperature (systemic elevation) and local muscle temperature, as the latter is more directly linked to improved contractile properties, nerve conduction velocity, and explosive performance [3]. Immediately after completing this phase and following the pre-test, participants were randomly assigned to one of three specific warm-up protocols:

1 **Dynamic Stretching (DS) Group**

Participants in this group performed eight consecutive dynamic exercises targeting active engagement of the lower limb muscles within functional ranges of motion. The exercises included: straight leg kicks, back kicks, butt kicks, high knee skipping, knee-to-chest stretch, leg cradles, karaoke steps to the right and left, and walking lunges. Each exercise was performed for 20 seconds in two sets (approximately 10–15 repetitions per set, depending on individual cadence), with 15 seconds of rest between sets and 30 seconds of rest between different exercises [25].

2 **TheraBand (TB) Group**

The TheraBand group followed the same sequence and exercise selection as the dynamic stretching protocol; however, all movements were performed with added resistance using elastic TheraBand bands (a black, hard resistance band, tension of approximately 4–6 kg at 100% stretch). For lower-body dynamic warm-up exercises, including walking lunges, high-knee skipping, and leg swings, elastic resistance bands were positioned around the thigh [26,27]. The use of TheraBand aimed to increase the mechanical load on the muscles and provide enhanced neuromuscular stimulation during the exercises. The duration, number of sets, rest intervals, and order of exercises were identical to those of the dynamic stretching group.

3 **Foam Roller (FR) Group**

Participants assigned to this group engaged in myofascial release exercises using a standard foam roller applied to four major muscle groups: gluteals, hamstrings, quadriceps, and gastrocnemius. This protocol follows previously established guidelines for foam rolling as a self-myofascial release technique [8,9,28,29]. Each muscle group was targeted in three sets of 30 seconds, with 15 seconds of rest between sets and 30 seconds between different muscle groups. Participants were instructed to apply pressure at a level sufficient to maintain effective contact with the fascial tissue without causing discomfort.

All protocols were conducted under the supervision of a trained researcher and adhered strictly to safety guidelines. Exercise intensity was regulated according to the Borg Rate of Perceived Exertion scale (RPE 6–8), ensuring consistency of effort across participants.

To enhance sport specificity, additional Taekwondo-relevant drills were included in both the dynamic stretching and TheraBand groups. For the Dynamic Stretching (DS) protocol, two movements simulating kicking patterns were added: front leg swings mimicking Ap Chagi and rotational hip drills replicating Dollyo Chagi. For the TheraBand (TB) group, resisted front kicks and lateral hip abduction exercises were incorporated using elastic bands to simulate Taekwondo kicking under load. The Foam Roller (FR) group followed standard myofascial release techniques as originally described. These modifications ensured that the interventions were more representative of Taekwondo’s biomechanical demands.

### Statistical analysis methods

All statistical analyses were conducted using SPSS software version 26. Data are presented as mean ± standard deviation for normally distributed variables and as median (interquartile range) for non-normally distributed variables. The level of statistical significance was set at p < 0.05. Normality of the dependent variables was assessed using the Shapiro–Wilk test for each group and time point. Homogeneity of variances was examined using Levene’s test. The assumption of homogeneity of regression slopes was verified by testing the interaction between the covariate (pre-test value) and group prior to the main analysis. For variables that met parametric assumptions, a one-way analysis of covariance (ANCOVA) was conducted with group (Foam roll, Dynamic, Theraband) as the between-subject factor, post-test values as the dependent variable, and pre-test values as the covariate. When normality or variance homogeneity assumptions were violated, a rank-based Quade’s ANCOVA was applied. In this procedure, post-test values were first adjusted for the covariate using linear regression, the residuals were ranked, and the ranked values were subsequently entered into a one-way ANOVA model to assess between-group differences.

For significant main effects, pairwise comparisons were performed using Bonferroni-adjusted post hoc tests for parametric data and Dunn–Bonferroni tests for rank-based analyses. Within-group pre–post changes were analyzed using paired-samples t-tests for normally distributed variables and Wilcoxon signed-rank tests for non-normally distributed variables.

## Results

Table 4 summarizes the kinetic and kinematic outcomes of the drop jump before (pre-test) and after the warm-up interventions. Baseline differences in eccentric phase duration (ECC Duration) and eccentric impulse were adjusted using rank-based ANCOVA, ensuring post-test comparisons reflect the effects of warm-up interventions rather than pre-existing disparities.Between-group comparisons showed no significant differences for jump height, flight time, contact time, RSImod, time to peak force, time to peak power, or eccentric impulse (p > 0.05), with trivial effect sizes (η² ≤ 0.104). In contrast, significant group effects were found for concentric phase duration (CON Duration; p = 0.040, η² = 0.177), peak concentric force (Peak Force CON; p = 0.034, η² = 0.186), and mean concentric power (Mean Power CON; p = 0.020, η² = 0.211).

**Table 4:** Kinetic and kinematic parameters during the drop jump task.

Task		Foam Roller	Dynamic	TheraBand	Effect size	p-value
jump height (m)	Pre	0.25 ± 0.06	0.25 ± 0.05	0.23 ± 0.05	0.029	0.627
	Post	0.24 ± 0.05	0.24 ± 0.04	0.22 ± 0.05		
Flight time (s)	Pre	0.43 (0.4, 0.49)	0.46 (0.41, 0.50)	0.43 (0.38, 0.43)	0.019	0.734
	Post	0.44 (0.41, 0.48)	0.43 (0.41, 0.48)	0.42 (0.38, 0.47)		
contact time (s)	Pre	0.42 ± 0.11	0.41 ± 0.09	0.40 ± 0.18	0.113	0.147
	Post	0.38 ± 0.10	0.38 ± 0.11	0.46 ± 0.18		
RSImod	Pre	0.55 (0.48, 0.77)	0.59 (0.51, 0.77)	0.69 (0.49, 0.77)	0.104	0.164
	Post	0.56 (0.5, 0.87)	0.65 (0.53, 0.76)	0.48 (0.41, 0.68)		
ECC Duration (s)	Pre	0.14 (0.10, 0.19)	0.19 (0.18, 0.20)	0.13 (0.08, 0.14)	0.011	0.838
	Post	0.15 (0.12, 0.2)	0.16 (0.12,0.18)	0.13 (0.08, 0.18)		
CON Duration (s)	Pre	0.22 (0.17, 0.39)	0.20 (0.18,0.24)	0.22 (0.12, 0.39)	0.177	0.040*
	Post	0.19 (0.14, 0.27)	0.19 (0.14, 0.26)	0.28 (0.18, 0.49)		
Time to Peak Force (s)	Pre	0.03 (0.03, 0.04)	0.03 (0.03, 0.04)	0.03 (0.03, 0.04)	0.008	0.880
	Post	0.03 (0.03, 0.04)	0.03 (0.03, 0.03)	0.03 (0.03, 0.04)		
Time to Peak Power (s)	Pre	0.39 (0.28, 0.49)	0.37 (0.32, 0.41)	0.31 (0.22, 0.51)	0.112	0.141
	Post	0.32 (0.27, 0.43)	0.33 (0.27, 0.36)	0.41 (0.27, 0.53)		
peak force con (N/BW)	Pre	5.04 (3.88, 5.94)	4.33 (3.93, 6.27)	5.10 (4.26, 6.55)	0.186	0.034*
	Post	4.48 (4.13, 5.83)	5.26 (4.7, 7.13)	5.86 (5.02, 7.66)		
peak power con (W/KG)	Pre	44.65 (36.17, 77.9)	40.89 (33.21, 57.84)	48.43 (38.73, 77.12)	0.083	0.239
	Post	39.97 (34.9, 66.42)	47.34 (30.19, 66.68)	52.81 (32.84, 65.55)		
mean power con (W/KG)	Pre	22.66 (17.05, 30.06)	23.63 (18.73, 28.92)	25.09 (17.68, 31.38)	0.211	0.020*
	Post	25.75 (20.59, 32.83)	25.05 (19.78, 35.22)	20.01 (11.97, 26.77)		
peak power ECC (W/KG)	Pre	−0.36 (−0.58,-0.15)	−0.22 (−0.33,-0.16)	−0.21 (−0.63, 0.15)	0.087	0.223
	Post	−0.44 (−0.67, −0.2)	−0.20 (−0.55, −0.08)	−0.23 (−0.44,-0.14)		
mean power ECC (W/KG)	Pre	−30.65 (−41.67, −24.9)	−32.82 (−3874, −26.56)	−37.57 (−54.82, −32.21)	0.043	0.485
	Post	−34.28 (−41.62, −29.85)	−29.95 (−42.21, −23.16)	−39.36 (−58.57, −33.77)		
Normalized ECC Impulse (S)	Pre	0.25 (0.22, 0.27)	0.28 (0.26, 0.31)	0.25 (0.24, 0.27)	0.053	0.407
	Post	0.26 (0.25, 0.29)	0.25 (0.23, 0.28)	0.26 (0.25, 0.29)		
Normalized CON Impulse (S)	Pre	0.22 (0.20, 0.29)	0.23 (0.19, 0.28)	0.24 (0.21, 0.29)	0.079	0.256
	Post	0.23 (0.2, 0.28)	0.24 (0.18, 0.27)	0.24 (0.19, 0.26)		

Data are expressed as mean ± standard deviation for normally distributed variables and as median (interquartile range) for non-normally distributed variables.* Statistically significant at the 0.05 level (p < 0.05).

Post-hoc Wilcoxon rank-sum tests indicated significant differences primarily between foam roller and TheraBand (CON Duration p = 0.015; Peak Force CON p = 0.024; Mean Power CON p = 0.013) and for Peak Force CON between foam roller and dynamic stretching (p = 0.043), as well as Mean Power CON between dynamic stretching and TheraBand (p = 0.028). Within-group analyses confirmed that foam rolling altered CON Duration (p = 0.026) and time to peak power (p = 0.041), dynamic stretching increased eccentric duration (p = 0.020) and enhanced Peak Force CON (p = 0.010) and Mean Power CON (p = 0.023), while TheraBand showed no significant changes.

**Fig 1:**
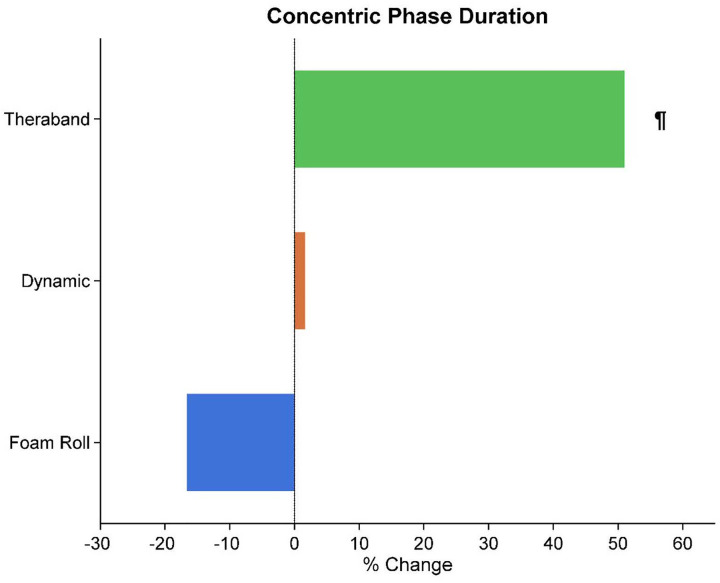
Concentric Duration % change. Within-group percentage changes during the drop jump were calculated as ((Post − Pre) / Pre) × 100. Foamroll (−16.63%), Dynamic (1.6%), TheraBand (50.94%). ¶: significant defference between TheraBand and FomRoller. P < 0.05.

**Fig 2:**
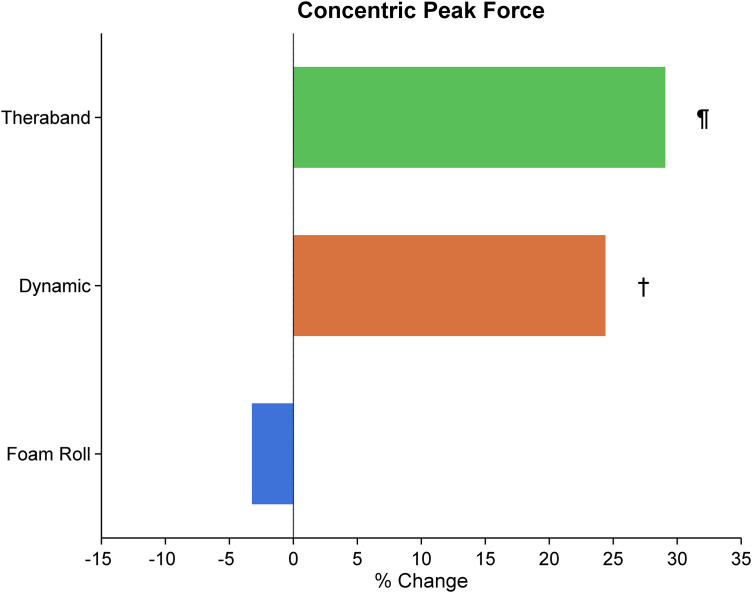
Concentric Peak Force % change. Within-group percentage changes during the drop jump were calculated as ((Post − Pre) / Pre) × 100. Foamroll (−3.22%), Dynamic (24.38%), TheraBand (29.06%). ¶: significant defference between TheraBand and FomRoller; †: significant defference between Dynamic and FomRoller. P < 0.05.

**Fig 3:**
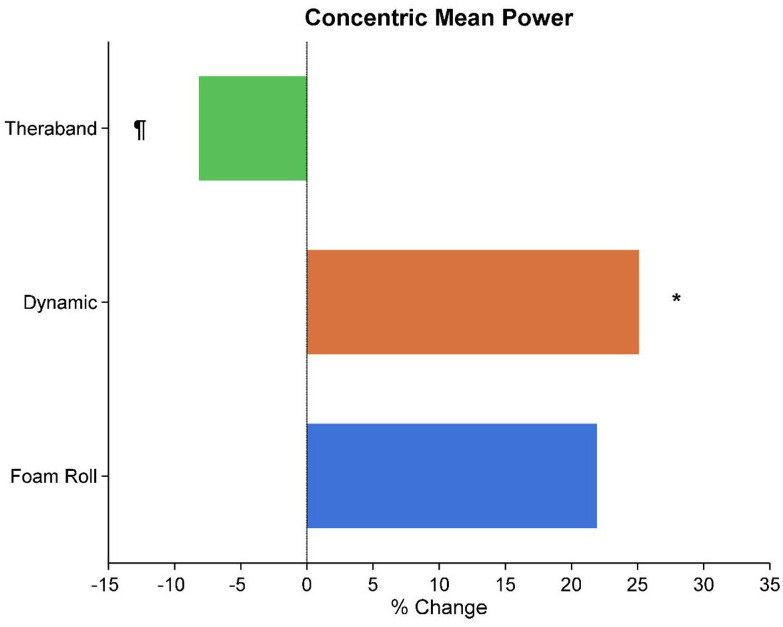
Concentric Mean Power % change. Within-group percentage changes during the drop jump were calculated as ((Post − Pre) / Pre) × 100. Foamroll (21.94%), Dynamic (25.11%), TheraBand (−8.15%). ¶: significant defference between TheraBand and FomRoller; *: significant defference between TheraBand and Dynamic. P < 0.05.

The percentage changes in concentric duration, mean concentric power and concentric peak force from pre-test to post-test across the different groups during the drop jump are illustrated in Figs 1–3.

As illustrated in Figs 1-3, Distinct response patterns were observed across the three warm-up protocols. Foam rolling was associated with reductions in concentric duration and peak force, whereas dynamic stretching improved peak force and mean power. Theraband exercise elicited the greatest increases in concentric duration and peak force but was accompanied by a reduction in concentric mean power.

## Discussion

Although the drop jump is not a direct component of Taekwondo competition, it provides a standardized biomechanical model to evaluate explosive lower-limb function similar to that required during kicking and landing maneuvers. The observed neuromechanical responses following the three warm-up protocols offer insight into how preparatory strategies acutely influence stretch–shortening cycle (SSC) behavior. Future research should apply these protocols during sport-specific kicking tasks to determine their transferability to competitive performance metrics, such as kick velocity and impact force. Beyond gross outcomes like jump height, recent biomechanical models emphasize that explosive performance is more strongly governed by the temporal organization of force production—including rate of force development (RFD), power–time slope, and phase coupling within the SSC—rather than peak force magnitude alone [30,31]. This framework aligns with the present findings, which demonstrate selective modulation of force–time variables without corresponding changes in jump height.

The present study examined the acute effects of foam rolling, dynamic stretching, and resistance-band exercises on drop jump kinetic and kinematic variables in elite Taekwondo athletes. The results showed that different warm-up modalities selectively influence variables related to force and power, with dynamic stretching selectively enhancing propulsive-phase mechanics. In contrast, overall jump outcomes such as height and flight time remained largely unaffected, highlighting the sensitivity of temporal and kinetic features of the stretch-shortening cycle to preparatory strategies. After controlling for baseline differences using ranked ANCOVA, significant group effects were observed for concentric phase duration (p = 0.040), peak concentric force (p = 0.034), and mean concentric power (p = 0.020). Effect size analysis indicated that the most substantial acute responses occurred in force- and power-related measures, whereas gross jump outcomes such as jump height (η² = 0.029) and flight time (η² = 0.019) exhibited minimal changes. Moderate effect sizes for peak concentric force (η² = 0.186) and mean concentric power (η² = 0.211) underscore that dynamic stretching selectively enhances propulsive-phase mechanics, whereas trivial effect sizes for gross outcomes reinforce the sensitivity of temporal and kinetic features to warm-up interventions.

Post-hoc comparisons revealed that significant differences occurred primarily between foam roller and TheraBand (CON Duration, Peak Force CON, Mean Power CON) and between dynamic stretching and TheraBand (Mean Power CON), supporting the selective modulation of propulsive-phase mechanics.

Two pre-test variables—eccentric duration and eccentric impulse—initially differed between groups but were statistically adjusted in the covariance model, confirming that post-test differences reflect warm-up effects rather than baseline disparities. Foam rolling elicited significant changes in concentric duration (p = 0.026) and time to peak power (p = 0.041), suggesting transient delays in force transmission and reduced neuromuscular responsiveness. These temporal alterations are consistent with the hypothesis that myofascial release acutely decreases muscle–tendon stiffness and elastic recoil efficiency, potentially affecting elastic energy transfer during SSC tasks [28]. However, some studies report negligible or inconsistent effects of foam rolling on neuromuscular performance, likely dependent on application duration, pressure, and target muscle group [8,29].

Dynamic stretching significantly increased eccentric duration (p = 0.020) and enhanced peak concentric force (p = 0.010) and peak concentric power (p = 0.023), supporting post-activation potentiation (PAP), whereby dynamic activation enhances neural drive and rate of force development during subsequent explosive tasks [32]. In contrast, the TheraBand protocol produced no significant within-group changes, indicating a neutral acute effect under the current protocol.

These differentiated responses suggest that warm-up strategies primarily modulate SSC function through temporal coordination and force-generating mechanisms rather than gross outcomes such as jump height or flight time. The dissociation between temporal adaptations and force magnitude aligns with evidence that SSC efficiency is dictated by inter-phase coordination, particularly the coupling between eccentric braking and concentric propulsion, rather than isolated peak outputs [33,34].

The observed lack of significant between-group differences for jump height, flight time, contact time, RSImod, eccentric duration, and time to peak force/power aligns with previous reports indicating that brief foam rolling or stretching does not necessarily impair jump height or power output [9,35,36]. These findings support the notion that elite neuromuscular systems exhibit resilience to short-term preparatory perturbations [1,2]. By ‘resilience,’ we refer to the ability of well-trained athletes to maintain overall performance outcomes (such as jump height and flight time) despite measurable changes in underlying biomechanical parameters (e.g., phase durations, force production patterns). This suggests that the neuromuscular system of elite athletes can adapt to and compensate for acute alterations in preparatory strategies without compromising gross functional output.

From a motor control perspective, alterations in power–time and force–time profiles likely reflect changes in motor unit recruitment, synchronization, and neuromuscular excitability, all of which are sensitive to warm-up modality, intensity, and contraction velocity. Dynamic warm-ups may enhance motor unit recruitment and synchronization, facilitating rapid force expression, whereas foam rolling and self-myofascial release can transiently modulate reflex sensitivity and neuromuscular responsiveness at the muscle spindle and corticospinal levels [37,38]. These results are consistent with Konrad et al. (2021) [39] and Fatahi et al. (2024) [40], who demonstrated that active and resistance-based warm-ups improve neuromuscular readiness and concentric power, whereas foam rolling may temporarily reduce muscle–tendon stiffness and delay power expression [8,29].

The selective alterations in eccentric and concentric timing, alongside changes in peak and mean concentric outputs, indicate that warm-up interventions modulate SSC efficiency through phase coordination rather than overall force magnitude. Prolonged eccentric duration observed in the dynamic group may reflect enhanced force absorption and elastic energy storage, whereas delayed concentric execution following foam rolling may compromise power transfer efficiency [34]. Even small temporal delays on the order of milliseconds can substantially reduce concentric power despite unchanged peak force, reinforcing the critical role of amortization efficiency and elastic energy transfer in explosive performance [41,42].In summary, after adjusting for baseline differences, dynamic stretching emerged as the most neuromechanically favorable warm-up, enhancing concentric force and power while optimizing phase timing. Foam rolling induced temporal modifications indicative of reduced neuromuscular responsiveness, whereas the TheraBand intervention exhibited neutral acute effects. Despite the large mean increase in concentric duration for the TheraBand group (58%), the high variability within this group indicates substantial inter-individual differences, which likely contributed to the lack of statistical significance in pairwise comparisons with the Dynamic group. Collectively, these findings highlight that warm-up modality primarily influences performance through force–time structure and SSC regulation rather than gross jump outcomes, with implications for targeted pre-performance preparation in elite athletes.

From a physiological perspective, the observed enhancements following dynamic stretching may be explained by the skeletal muscle pump mechanism. Dynamic warm-up likely enhances muscle perfusion through rhythmic contractions that increase venous return and arterial blood flow. This heightened vascularization leads to increased local muscle temperature, which in turn improves muscle contractile properties, nerve conduction velocity, and metabolic enzyme activity [3]. These physiological adaptations are particularly beneficial for brief, explosive actions such as Taekwondo kicks, which require rapid force development and relaxation.

It should be noted that the present findings are limited by the small sample of elite female athletes, the use of drop jump as a proxy for sport-specific kicking actions, and the acute nature of the interventions. Therefore, caution is advised when generalizing these results to other populations, longer-term training effects, or competitive performance outcomes.

### Practical implications

These results suggest that evaluating temporal and force–time characteristics can complement traditional measures, such as jump height, when assessing warm-up effects in elite Taekwondo athletes. Dynamic stretching was linked to increased concentric force and power, foam rolling to subtle changes in SSC timing, and resistance-band exercises generally maintained baseline performance. Coaches may consider matching warm-up modality to session goals: dynamic stretching for rapid force expression, foam rolling for flexibility or recovery-focused sessions, and resistance bands to preserve readiness. These recommendations reflect acute effects only; long-term adaptations, recovery, and injury risk were not assessed. Nonetheless, this study provides a cautious, evidence-informed framework to guide warm-up selection and support practical decision-making in high-performance training.

## Conclusion

The present study indicates that acute warm-up interventions elicit modality-specific neuromechanical responses in elite Taekwondo athletes. Dynamic stretching was associated with modest enhancements in eccentric–concentric coordination and concentric power, while foam rolling primarily affected temporal aspects of force and power development without consistently increasing peak outputs. Resistance-band exercises largely maintained baseline performance.These results suggest that changes in force–time and power–time characteristics may provide a more sensitive measure of acute readiness than traditional gross outcomes such as jump height. The dissociation between output magnitude and temporal adaptations underscores the relevance of phase coordination for explosive movements.Overall, warm-up strategies should be selected based on the specific neuromechanical demands of the task. Further research is warranted to determine whether these acute effects translate into improvements in sport-specific skills or long-term neuromuscular adaptations.

## Supporting information

S1 Dataset Dataset. (XLSX)

S1 File Image S1. (JPG)

S2 File Image S2. (JPG)
