# Supplementary figures and images for: Acute effects of three warm-up protocols on drop jump biomechanics in elite Taekwondo athletes: An IMU-based analysis
Source: PLoS One. 2026 Jun 22;21(6):e0351884. doi: 10.1371/journal.pone.0351884 (PMC13286194; doi:10.1371/journal.pone.0351884)

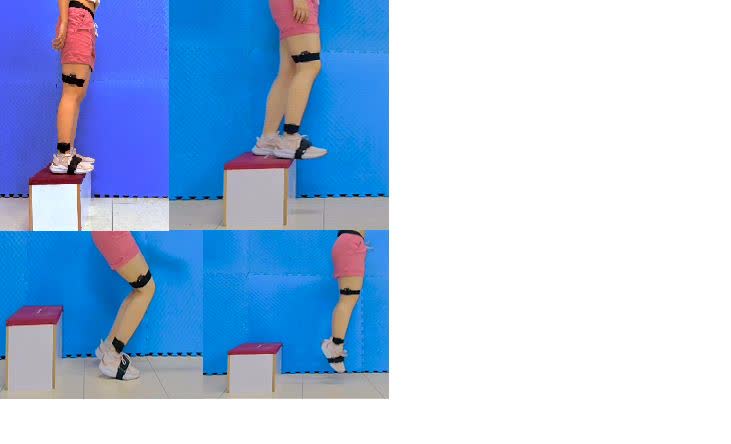

Supplement: S1 File — (JPG) [file pone.0351884.s002.jpg]

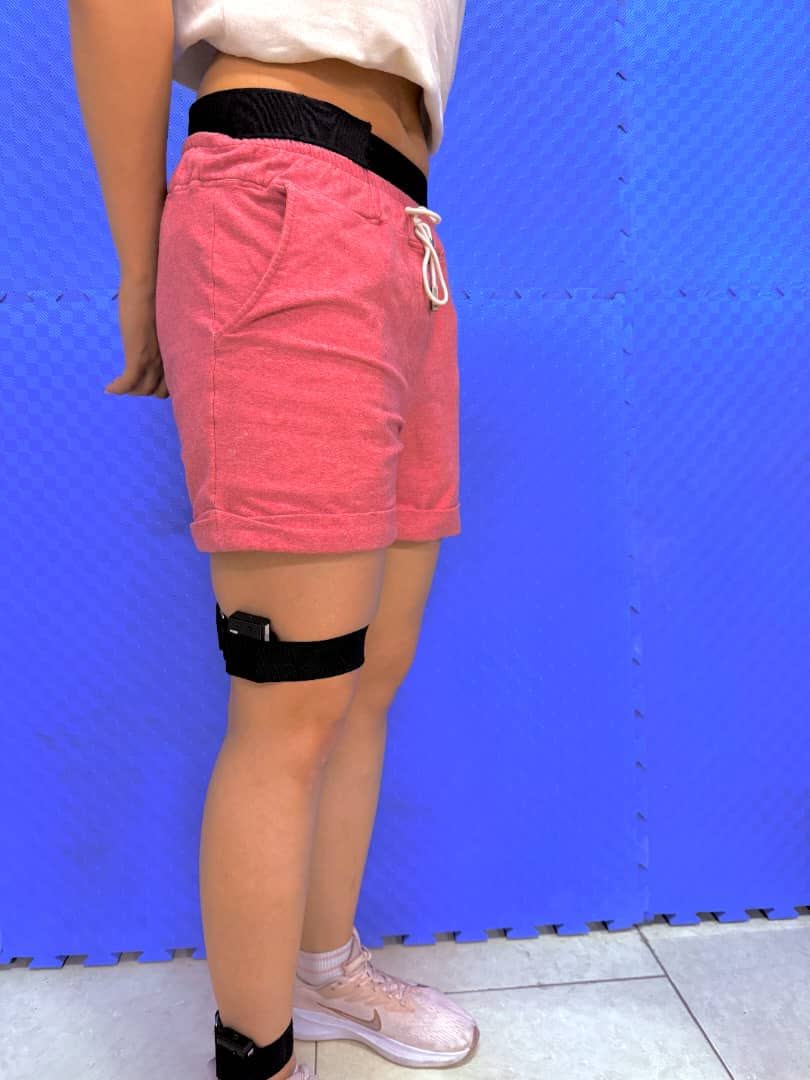

Supplement: S2 File — (JPG) [file pone.0351884.s003.jpg]
